# High-dose accelerated intermittent theta burst stimulation targeting the primary motor cortex for gait and cognitive functions in cerebral small vessel disease: a randomized controlled trial

**DOI:** 10.3389/fneur.2026.1840684

**Published:** 2026-06-01

**Authors:** Xiao-Ying Zhao, Jia-Jing Tian, Bei-Yan Guan, Hua-Wei Chen, Shi-Yao Wang, Xin-Yang Zhang, Yan-Wei Miao, Chun-Li Song, Bing-Wei Zhang

**Affiliations:** 1 Department of Neurology, The First Affiliated Hospital of Dalian Medical University, Dalian, China; 2 Department of Convalescent Medicine IV, Dalian Rehabilitation Recuperation Center of Joint Logistics Support Force of PLA, Dalian, China; 3 Department of Radiology, The First Affiliated Hospital of Dalian Medical University, Dalian, China

**Keywords:** accelerated intermittent theta burst stimulation, cerebral small vessel disease, cognitive dysfunction, gait disorder, neuroimaging phenotype, primary motor cortex

## Abstract

**Background:**

Gait disorder and cognitive dysfunction are the most common symptoms in patients with cerebral small vessel disease (CSVD), significantly impacting patients’ quality of life. Currently, there remains a lack of effective treatment for gait disorder and cognitive dysfunction of CSVD. In this randomized, single-blind, sham-controlled study, we conducted high-dose accelerated intermittent theta burst stimulation (aiTBS) in 36 patients with CSVD to investigate the efficacy and safety of high-dose aiTBS targeting the primary motor cortex (M1 area) for treating various symptoms of CSVD, particularly gait and cognitive function.

**Methods:**

The patients were randomly assigned to two groups of real (*n* = 19) or sham (*n* = 17) aiTBS targeting the primary motor cortex. Both groups received 14 consecutive sessions of real-aiTBS or sham-aiTBS. Primary outcome was the change of 3-meter Timed Up and Go (3mTUG) duration, assessed at baseline (T0) and immediately post-intervention (T1), with follow-up evaluations at 4 weeks after intervention (T2). Secondary outcomes included changes in the Tinetti Performance-Oriented Mobility Assessment (Tinetti) score, the Chinese version of the Mini-Mental State Examination (CMMS) score, the Montreal Cognitive Assessment (MoCA) score, three-dimensional gait analysis, and multidimensional function scale scores after intervention.

**Results:**

Compared to the sham-aiTBS group, the real-aiTBS group exhibited significantly greater improvements in multidimensional gait, cognitive, affective and autonomic nervous function assessments. At the 4-week follow-up, time effects were statistically significant for the 3mTUG duration, Tinetti, CMMS, and MoCA scores. The real-aiTBS group exhibited more pronounced group-by-time interaction effects for the 3mTUG duration, Tinetti, and CMMS scores, while no statistically significant differences from the sham-aiTBS group were observed for the MoCA score. The aiTBS intervention response is correlated to the CSVD neuroimaging features, including periventricular white matter hyperintensity, enlarged perivascular space, cortical atrophy, lacune and total CSVD burden score.

**Conclusion:**

The aiTBS holds promise as a valuable therapeutic approach for CSVD. High-dose aiTBS targeting the M1 area improved clinical symptoms such as gait and cognitive disorder in patients with CSVD. The therapeutic response to aiTBS in CSVD patients is related to the CSVD neuroimaging phenotypes.

## 1 Introduction

Cerebral Small Vessel Disease (CSVD) refers to a group of clinical, neuroimaging, and pathological manifestations resulting from damage to the cerebral microcirculation. Clinically, CSVD is commonly associated with cognitive impairment, gait disturbance, affective disorder, and autonomic dysfunction (1). With the rapid progress of population aging, the morbidity of CSVD continues to rise, imposing a substantial burden on patients and healthcare systems (2). Among its various manifestations, gait disorder is a prominent and disabling feature, characterized by impaired balance and locomotor function, which contributes to long-term functional decline and markedly increases the risk of falls and other adverse outcomes (3).

Current management of CSVD primarily focuses on pharmacologic control of traditional vascular risk factors and lifestyle modification (4). However, treatment approaches for CSVD-related gait disorder remain limited in clinical practice. Rehabilitation is generally considered the primary method, including gait and balance training, as well as multimodal programs integrating physical exercise with cognitive training (5). Nonetheless, treatment efficacy is often constrained by compliance and baseline motor ability. Although Acetylcholinesterase inhibitors, the most commonly used anti-dementia drugs at present, have been tried in the treatment of cognitive impairment in patients with CSVD, their potential limitations and long-term efficacy still require further research (6). Therefore, there is an urgent need for novel interventions to better preserve mobility and slow the progression of gait disorder and cognitive dysfunction in patients with CSVD.

Noninvasive brain stimulation (NIBS) is a technique that modulates the excitability of specific brain regions and functional networks (7). As one of NIBS paradigms, transcranial magnetic stimulation (TMS) induces focal electric currents in cortical tissue via rapidly changing magnetic fields, thereby altering cortical excitability and network activity (8). Although the underlying mechanisms are not fully elucidated, accumulating evidence suggests that TMS may influence neuroplasticity, electrophysiological dynamics, and neurotransmitter release (9–11). Clinically, TMS has demonstrated therapeutic benefits in some neurological and psychiatric disorders, including Parkinson’s disease, Alzheimer’s disease, and treatment-resistant depression (12–14). Normal gait control relies on the coordinated function of multiple circuits, including cortico–basal ganglia–thalamic and cortico-cerebellar loops, frontoparietal–limbic networks, and the corticospinal tract (15–17). CSVD-related gait disorder is plausibly driven by microvascular injury that disrupts these important pathways and impairs network integration. Notably, in Parkinson’s disease, TMS has been shown to improve motor symptoms by modulating functional connectivity and topological organization within key motor-control circuits (18–20). These findings support the rationale that TMS represents a promising non-pharmacological approach to neuromodulate network dysfunction in CSVD and ultimately improve gait disorder.

Theta burst stimulation is a specialized mode of TMS, categorized based on stimulation interval duration: intermittent theta-burst stimulation (iTBS) enhances cortical excitability, while continuous theta-burst stimulation (cTBS) reduces it (9). Accelerated intermittent theta burst stimulation (aiTBS) refers to administering two or more iTBS interventions within a single day (21). This accelerated protocol can substantially shorten the overall treatment course, allow more flexible session planning, and potentially improve adherence, with favorable safety and tolerability reported (22). Moreover, within a defined range, the total number of stimulation pulses appears to exert a dose-dependent effect on excitability enhancement (23). The M1 area (primary motor cortex, Brodmann area 4) was selected as the stimulation target. Beyond motor control, M1 area also contributes to higher-order cognitive processes through its extensive connections with prefrontal and subcortical regions (24). As a key hub within the motor network, M1 area integrates sensory, cognitive, and motor inputs to support postural control and coordinated gait (25, 26). Consistent with this network-based role, TMS targeting M1 area has been reported to alleviate motor symptoms, including gait impairment in Parkinson’s disease, potentially via concomitant modulation of cortical excitability and large-scale functional connectivity across distributed motor-related circuits (27–29).

This study employs a randomized sham-stimulation controlled design to investigate the therapeutic efficacy and safety of high-dose aiTBS targeting M1 area for functional abnormalities such as gait disorder and cognitive dysfunction in CSVD patients. We hypothesized that CSVD patients in the real-aiTBS group will show better gait and cognitive function improvement compared to the sham-aiTBS group after one course of aiTBS.

## 2 Materials and methods

### 2.1 Participants

This study enrolled 36 CSVD patients who presented to The First Affiliated Hospital of Dalian Medical University from February 2023 to August 2025. The inclusion criteria were the following: (1) Confirmed CSVD diagnosis according to the Chinese Expert Consensus on Diagnosis and Treatment of Cerebral Small Vessel Disease 2021 (30). (2) Age range of 18–85 years. (3) Previously had not undergone TMS intervention. The exclusion criteria were the following: (1) Acute phase of large artery atherosclerotic, cardioembolic, or other rare etiologies of stroke. (2) Intracranial major vessel occlusion or stenosis >50% on imaging. (3) Neurological disorders affecting cognition or motor coordination (e.g., epilepsy, spinal cord disease, Parkinson’s disease, Alzheimer’s disease). (4) Psychiatric disorders. (5) History of central nervous system trauma (including cranial trauma or neurosurgical procedures) or severe systemic diseases. (6) Spinal, joint, musculoskeletal abnormalities or anatomical anomalies. (7) Contraindications for MRI or inability to cooperate with gait or neurological function assessments. (8) Contraindications for TMS.

All subjects were randomly assigned in a 1:1 ratio to receive real or sham aiTBS daily for 7 consecutive days. All subjects remained blind to the grouping. This study was conducted with ethical approval from the Institutional Ethics Committee of The First Affiliated Hospital of Dalian Medical University (Ethics code: PJ-KS-KY-2023-04(X)) and retrospectively registered in the Chinese Clinical Trial Registry (https://www.chictr.org.cn/; Unique identifier: ChiCTR2500109705). Informed consent was obtained from all participants prior to their participation.

Using G*Power 3.1 for a two-tailed t-test (*α* = 0.05, power = 0.80) and an expected effect size of Cohen’s *d* = 0.6 (31), we needed 39 participants per group (total 78). Accounting for 20% dropout, we aimed for 98. We enrolled 50 (25/group); with this, the minimum detectable effect size was Cohen’s *d* = 0.81 and achieved power for Cohen’s *d* = 0.64 was 79%. The completer sample (real-aiTBS group: *n* = 19; sham-aiTBS group: *n* = 17) gave an observed effect size of Cohen’s *d* = −1.659 and post-hoc power >0.999, though the primary justification remains the literature-derived effect size.

### 2.2 The aiTBS intervention

The aiTBS was administered using a Magstim Rapid2 transcranial magnetic stimulator (The Magstim Company Limited, UK). The aiTBS was delivered using a figure-8 coil centered at Cz (10–20 system), handle posterior, targeting the bilateral leg M1 area (paracentral lobule). The proximity of leg M1 to the midline (1–2 cm) and evidence that the optimal scalp site for lower-limb MEPs is near the midline (32) make this placement methodologically reasonable for bilateral influence. No left/right M1 distinction was made. All subject received 2 daily sessions with ≥4-h intervals over 7 consecutive days. Each session consisted of 67 bursts, each containing an 8 s-train of 37 Hz pulses followed by a 2 s interval, repeated continuously for 670 s to deliver a total of 2010 pulses. Stimulation intensity was set to 40% resting motor threshold (RMT) in the real-aiTBS group. The resting motor threshold was set to the minimum intensity that produced a ≥ 50 μV motor-evoked potential in the relaxed first dorsal interosseous muscle on at least 5 out of 10 trials. The sham-aiTBS group received the same stimulation protocol as the real-aiTBS group, except for 0% RMT intensity, with the recording device reproducing identical stimulation sounds on the scalp surface. All subjects received standard pharmacologic treatments during the intervention period, including antiplatelet therapy, hypolipidemic therapy, antihypertensive therapy, as recommended by the Chinese Expert Consensus on Diagnosis and Treatment of Cerebral Small Vessel Disease 2021 (30).

### 2.3 Primary outcome

The primary outcome was the change from baseline (T0) to follow-up (T2) in the 3-meter Timed Up-and-Go test (3mTUG) duration. The 3mTUG duration was selected as the sole primary endpoint because it is a direct, objective, and clinically feasible measure of functional mobility, which is the primary focus of this trial in CSVD patients, which was administered as follows: subjects stood from an armless chair on hearing the “go” command, walked at maximum speed along a 3-meter path, turned 180°, returned to the chair, and resumed seated position. The total time from standing to the moment the hip touched the seat was recorded. Each subject performed the test three times, with a 1-min rest between trials, and the average time of the three trials was used for analysis.

### 2.4 Secondary outcomes

**Figure 1:**
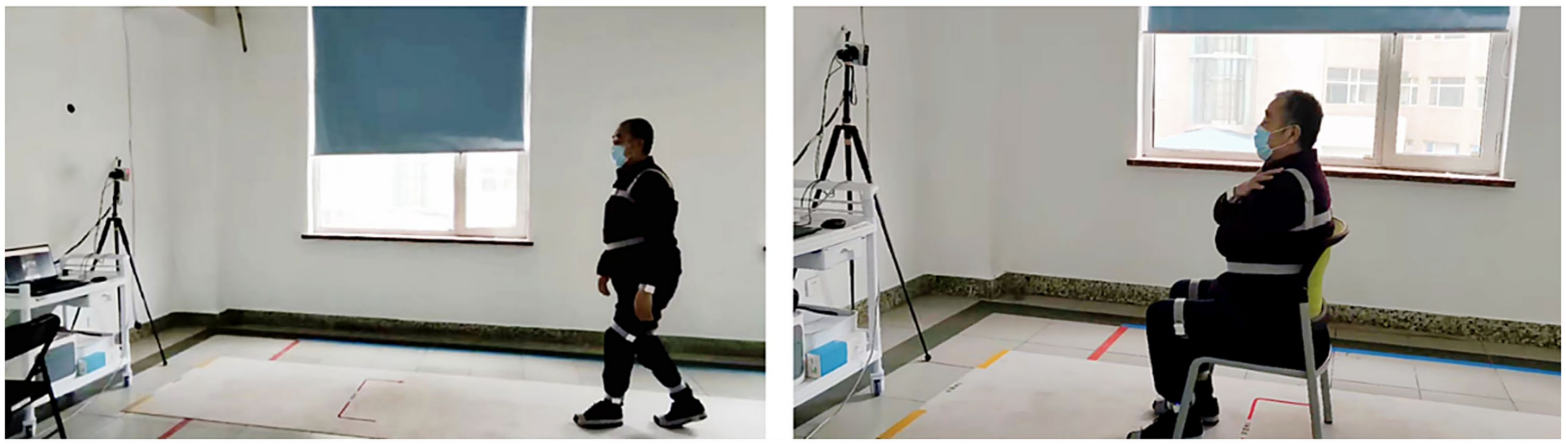
The process of gait assessment.

Secondary outcomes included the changes from baseline to post-intervention in the Tinetti Performance-Oriented Mobility Assessment (Tinetti), three-dimensional gait parameters and multidimensional neurological function assessments in both groups, especially focusing on changes the Chinese version of the Mini-Mental State Examination (CMMS) (33, 34) and the Montreal Cognitive Assessment (MoCA) for comprehensive cognitive ability evaluation. The three-dimensional gait analysis utilizes the MATRIX RuiPing V2.0 gait quantification assessment system, which using a wearable measurement unit system equipped with 10 sensors placed on the anterior chest, lower back, wrists, thighs, shanks, and feet. Each subject completed a series of standardized tests, including the 5-meter Timed Up and Go, standing with eyes closed, semi-tandem stance, tandem stance, and five-repetition sit-to-stand test. The following spatiotemporal gait parameters were extracted to evaluate fundamental pace characteristics: step length, stride length, walk speed and cadence. The gait cycle, swing, stride variability, stride velocity variability, and swing absolute difference were used to evaluate gait phasing and variability. Additionally, going straight trunk - sway max, close eyes trunk - sway max, mid-series trunk - sway max, and in-series trunk - sway max, as well as the average duration for chair stand, were analyzed to assess gait stability. The assessment process is shown in Figure 1. The Tinetti score comprises subscales for gait and balance, with a maximum score of 28. Lower scores indicate poorer gait and balance function.

A comprehensive function assessment battery was administered, which included the following instruments: the Hamilton Anxiety Scale (HAMA) and Hamilton Depression Scale (HAMD) for evaluating affective state. The Trail Making Test-A (TMT-A) and the Trail Making Test-B (TMT-B) to assess visuospatial ability and executive function. The Digital Span Test (DST) was used to measure immediate memory and attentional capacity, while the Boston Naming Test (BNT) was employed to assess language function. The Clock Drawing Test (CDT) was administered to examine visuospatial, executive, and graphomotor abilities. The Judgment of Line Orientation Test (JLO) was utilized to evaluate visuospatial perception. And the Overactive Bladder Symptom Score (OABSS) was applied to screen for the presence of voiding dysfunction.

Trained physicians who were unclear to all clinical data rated these brain MRI markers. Neuroimaging markers of CSVD were rated according to the Standards for Reporting Vascular Changes on Neuroimaging (STRIVE-2) criteria (35). White matter hyperintensities were assessed using the Fazekas scale. Enlarged perivascular spaces were evaluated using the Potter scale. Global cortical atrophy was rated using the global cortical atrophy scale. Deep brain lobe atrophy was assessed using the medial temporal lobe atrophy visual rating scale. The number of lacunar infarcts and cerebral microbleeds was counted.

The safety assessment mainly focused on the incidence of adverse events such as epilepsy, syncope, hearing impairment, neck pain, burning sensation and insomnia during the intervention of aiTBS.

### 2.5 Procedure

Subjects underwent comprehensive clinical examinations and brain magnetic resonance imaging scans, followed by screening based on predefined inclusion and exclusion criteria for enrollment. After baseline assessments (T0) including gait analysis and neurological function assessment, subjects received consecutive 7-day interventions of real or sham aiTBS. Immediately after the end of aiTBS (T1), gait assessment and neurological function assessment were performed again, which were the same as baseline. At 4 weeks after aiTBS (T2), follow-up evaluations were conducted for 3mTUG duration, Tinetti, CMMS and MoCA scores. The timing and content of all assessments are summarized in Table 1.

**Table 1:** Schedule of data collection time points and assessment contents.

Time point	Timing	Assessments (grouped by domain)
T0 (Baseline)	Day 0	Gait: 3mTUG, Tinetti, 3D gait analysis Cognition: CMMS, MoCA, DST, CDT, BNT, TMT, JLO Emotion: HAMA, HAMD Overactive bladder: OABSS
T1 (Post-intervention)	Day 7	Exactly the same as T0
T2 (Follow-up)	Week 5 (4 weeks after T1)	3mTUG, Tinetti, CMMS, MoCA

Key summary parameters are recorded in the CRF (Supplementary Table S5); the full set of parameters for all participants at T0 and T1 is provided in Supplementary Gait_Parameters_Template. 3mTUG, 3-meter Timed Up and Go; CMMS, Chinese version of the Mini-Mental State Examination; MoCA, Montreal Cognitive Assessment; DST, Digital Span Test; CDT, Clock Drawing Test; BNT, Boston Naming Test; TMT, Trail Making Test; JLO, Judgment of Line Orientation Test; HAMA, Hamilton Anxiety Scale; HAMD, Hamilton Depression Scale; OABSS, Overactive Bladder Symptom Score.

### 2.6 Statistical analysis

Continuous variables with normal distribution were described as mean ± standard deviation and analyzed using the independent samples t-test. Non-normally distributed continuous variables were summarized as median (interquartile range) and compared with the Mann–Whitney *U* test. Categorical variables were presented as frequency (percentage) and analyzed using the Fisher’s exact test. For longitudinal within-group comparisons of gait parameters and neurological function scale scores between baseline (T0) and immediately after aiTBS (T1), the Shapiro–Wilk test was first applied to the differences between T0 and T1 values. For measures where the differences followed a normal distribution, the paired samples *t*-test was used; otherwise, the Wilcoxon signed-rank test was applied. A generalized linear mixed model (GLMM) was employed to analyze the repeated measures data, which is appropriate for longitudinal study designs involving both within-subject repeated measurements and between-subject group differences. Baseline (T0) values were included as a covariate to control for potential confounding due to initial differences between groups and to improve internal validity. The model incorporated fixed effects for time (T0, T1, T2), group (real-aiTBS vs. sham-aiTBS), and the time-by-group interaction term. These terms were used to evaluate the overall temporal trend, group differences, and the interaction between group and time, respectively. The statistical significance of each effect was assessed using *F*-tests and corresponding *p*-values, with 95% confidence intervals reported to aid in the interpretation of effect sizes. To facilitate clinical interpretation, we calculated effect sizes for the primary Group × Time interactions. Partial η^2^ (η^2^_p_) was computed from the GLMM *F*-values and degrees of freedom using the formula: η^2^_p_ = $F\times df_{\text{effect}}/(F\times df_{\text{effect}}+df_{\text{error}})$ . Effect size was interpreted using the conventional thresholds: small if η^2^_p_ < 0.06, medium if 0.06 ≤ η^2^_p_ < 0.14, and large if η^2^_p_ ≥ 0.14. Values below 0.01 were considered negligible (36).

To explore which patient group exhibit better therapeutic efficacy, spearman correlation analyses were used to investigate the associations between baseline neuroimaging CSVD markers and post-treatment changes in gait and cognitive outcomes. A two-tailed test was used for all analyses, with statistical significance defined as *p* < 0.05.

## 3 Results

**Figure 2:**
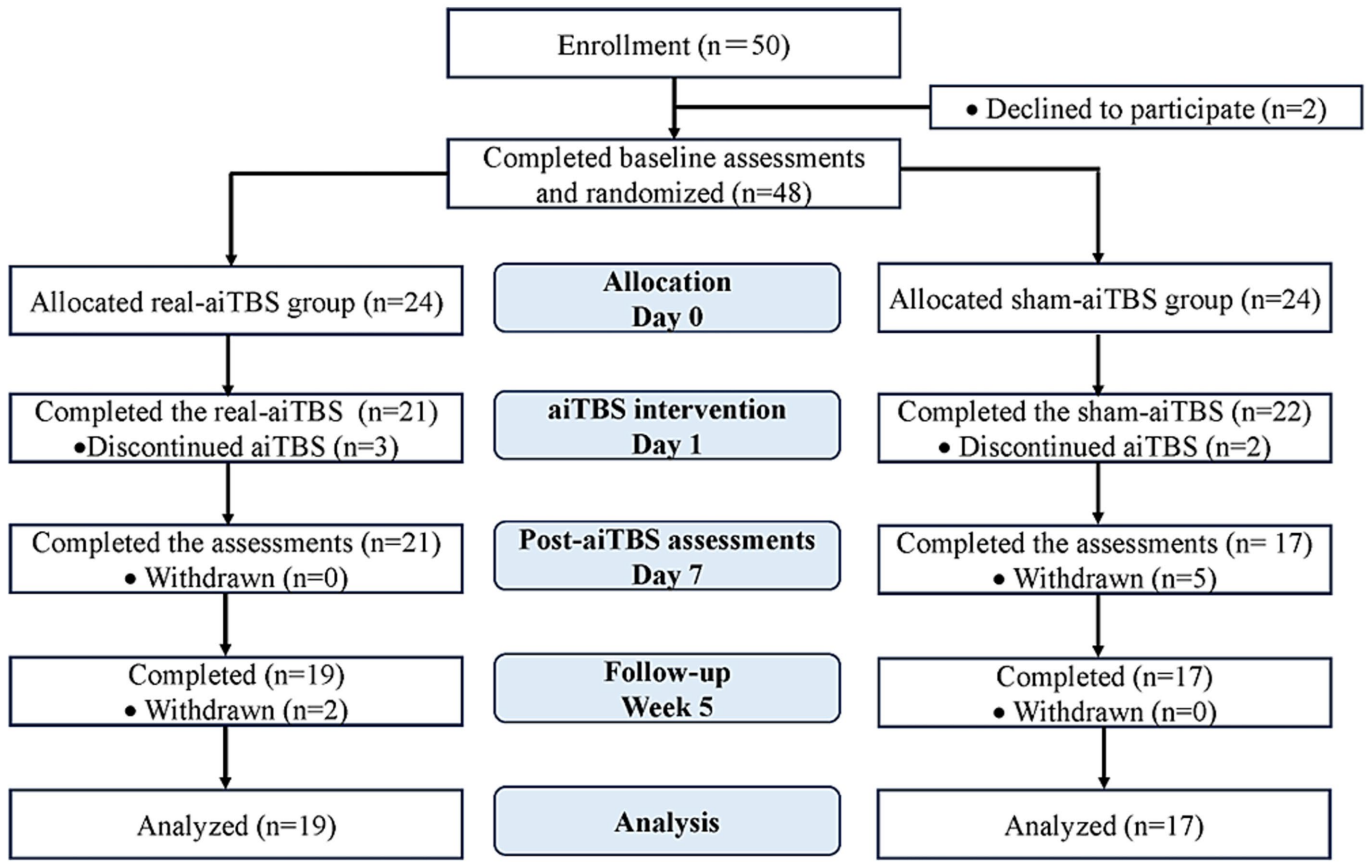
The flow diagram of this study.

From the initial enrollment of 50 subjects, 48 were successfully allocated to the real-aiTBS groups (*n* = 24) or sham-aiTBS groups (*n* = 24). During the intervention phase, three subjects in the real-aiTBS group and two in the sham-aiTBS group voluntarily discontinued the trial. At the immediate post-treatment, five participants in the sham-aiTBS group could not complete assessment due to personal reasons. Subsequently, two subjects in the real-aiTBS group were non-adherent to the follow-up visit. The final analysis included data from 36 participants who completed the entire study protocol (Figure 2). To address whether dropouts were related to the intervention or outcome, we have compared baseline characteristics between completers and dropouts (Supplementary Table S1), finding no significant differences (*Ps* > 0.05).

### 3.1 Characteristics of the study population

Baseline demographic and clinical characteristics were well-balanced between the two groups, with the exception of hypertension history (Table 2). The decision to match groups by total CSVD burden score was based on the considerable heterogeneity in neuroimaging presentations observed clinically in CSVD patients, wherein the severity of clinical symptoms is not consistently proportional to the total CSVD burden score (3).

**Table 2:** Demographic and clinical data at Baseline.

Outcome	Real-aiTBS Group (*n* = 19)	Sham-aiTBS Group (*n* = 17)	Statistic value	*P*-value
Age, Mean ± SD	69.58 ± 4.46	67.47 ± 7.69	*t* = 1.019	0.315
Gender (male/female)	12/7	9/8	–	0.736
Education, M (Q₁, Q₃)	10.00 (8.00, 15.00)	12.00 (9.00, 15.00)	*Z* = –0.468	0.640
BMI, M (Q₁, Q₃)	24.77 (22.90, 25.96)	26.00 (23.60, 27.05)	*Z* = –1.300	0.194
Smoking (%)	4/15 (21.05)	3/14 (17.65)	–	1.000
Drinking (%)	6/13 (31.58)	2/15 (11.76)	–	0.236
Stroke (%)	7/12 (36.84)	6/11(35.29)	–	1.000
Hypertension (%)	14/5 (73.68)	17/0 (100.00)	–	0.047*
Diabetes mellitus (%)	2/17 (10.53)	5/12 (29.41)	–	0.219
Atrial fibrillation (%)	1/18 (5.26)	0/17 (0.00)	–	1.000
Total CSVD burden score (%)			–	0.315
1	3 (15.79)	0 (0.00)		
2	3 (15.79)	4 (23.53)		
3	13 (68.42)	12 (70.59)		
4	0 (0.00)	1 (5.88)		

*P < 0.05.

### 3.2 Follow-up assessment

**Figure 3:**
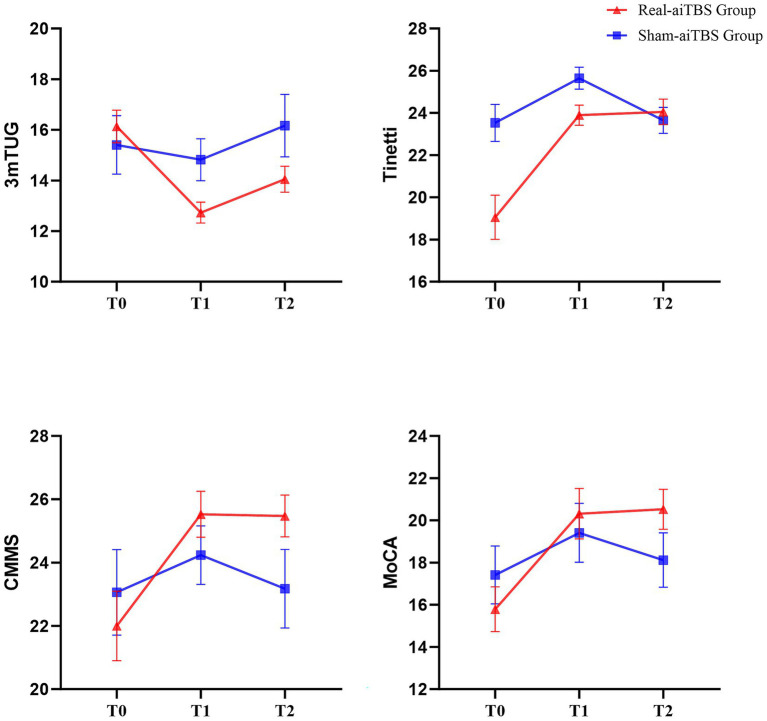
Comparison of follow-up results between two groups. aiTBS = accelerated intermittent theta burst stimulation; 3mTUG = 3-meter timed up and go; Tinetti = Tinetti performance-oriented mobility assessment; CMMS: Chinese version of the Mini-Mental State Examination; MoCA = Montreal Cognitive Assessment.

For the longitudinal assessment of gait and cognitive function across multiple time points, this study focused on four key metrics: the 3mTUG duration, Tinetti, CMMS and MoCA scores. The results regarding the main effects of time and group, as well as the time-by-group interaction, are summarized as follows: in the comparison of 3mTUG duration, these results indicate a general improvement over time, with a more pronounced reduction in 3mTUG duration in the real-aiTBS group. The evaluation of Tinetti score suggesting a time-dependent superiority of real-aiTBS group in improving Tinetti scores. The comparison of CMMS score indicating a steeper slope of improvement in the real-aiTBS group over time. The evaluation of MoCA suggesting that real-aiTBS did not confer a statistically significant advantage over sham-aiTBS in improving MoCA score (Table 3 and Figure 3).

**Table 3:** Generalized linear mixed model analysis of changes from T0 to T2.

Outcome	Estimated change (T2-T0)		Time effect		Group effect	Group × Time effect		
	Real-aiTBS group Mean [95%CI]	Sham-aiTBS group Mean [95%CI]	*F*-test	*P*-value	*F*-test	*P*-value	*F*-test	*P*-value
3mTUG	−2.08[−2.95,–1.21]	0.76 [0.09,1.44]	153.404	<0.001	17.191	<0.001	65.013	<0.001
Tinetti	5.00 [3.56,6.44]	0.12[−1.02,1.25]	20.552	<0.001	4.774	0.031	7.848	0.001
MMSE	3.47 [1.95,4.99]	0.12[−0.65,0.89]	52.107	<0.001	0.592	0.443	30.003	<0.001
MoCA	4.74 [3.02,6.46]	0.71[−0.60,2.01]	20.694	<0.001	2.749	0.100	2.198	0.116

The Group × Time interaction was significant for 3mTUG duration, Tinetti, and CMMS (*Ps* < 0.001). Effect sizes for these outcomes were medium to large, confirming the clinical relevance of the improvements in the real-aiTBS group (Table 4). For the MoCA, the interaction was not significant (*p* = 0.116, η^2^_p_ = 0.041).

**Table 4:** Effect sizes for Group × Time interaction (T2–T0 change).

Outcome	Partial η^2^	Interpretation
3mTUG	0.560	Large
Tinetti	0.133	Medium-to-large
CMMS	0.370	Large
MoCA	0.041	Small

### 3.3 Gait assessments

**Figure 4:**
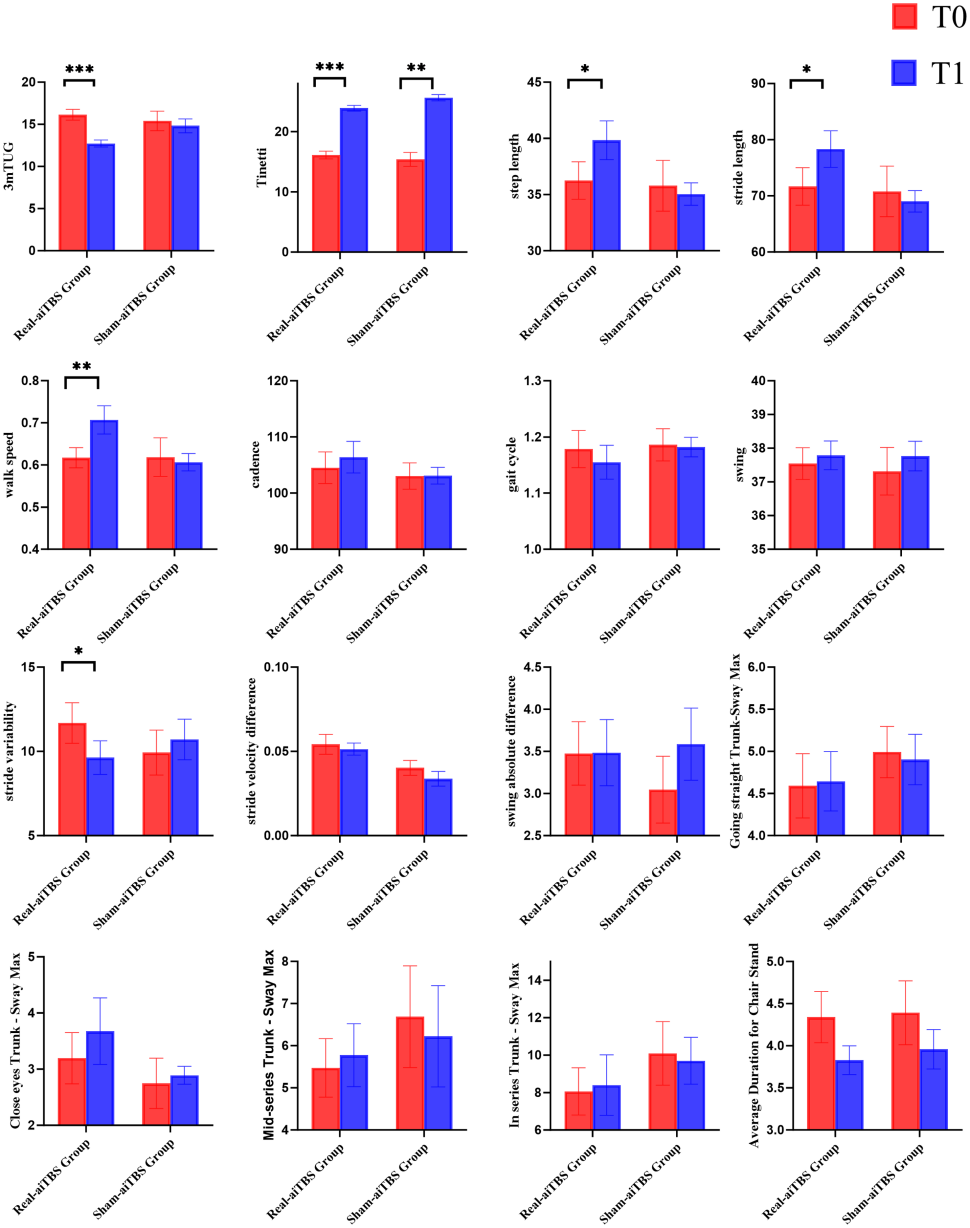
Comparison of gait parameters between two groups. *p < 0.05; ** *p* < 0.01, ***p < 0.001.

With the exception of the Tinetti score, the real and sham aiTBS groups demonstrated comparable baseline profiles across all gait parameters. In post-intervention assessment, within-group analyses indicated that real aiTBS resulted in significant improvements relative to baseline in the 3mTUG duration, Tinetti score, step length, stride length, walking speed, and stride variability (*P*s < 0.05). Conversely, a significant improvement in the sham-aiTBS group was confined to the Tinetti score (*p* < 0.05, Figure 4).

Although baseline Tinetti scores differed between groups, our primary GLMM adjusted for baseline values as pre-specified. To further confirm robustness, we conducted an ANCOVA with Tinetti change score (T2 − T0) as dependent variable, baseline Tinetti as covariate, and Group as fixed factor. After adjustment, the real-aiTBS group showed significantly greater improvement than the sham group (*F* = 17.951, *p* < 0.001, adjusted R^2^ = 0.801), confirming that the treatment effect is not an artifact of baseline imbalance (Supplementary Table S2).

### 3.4 Cognition function assessments

**Figure 5:**
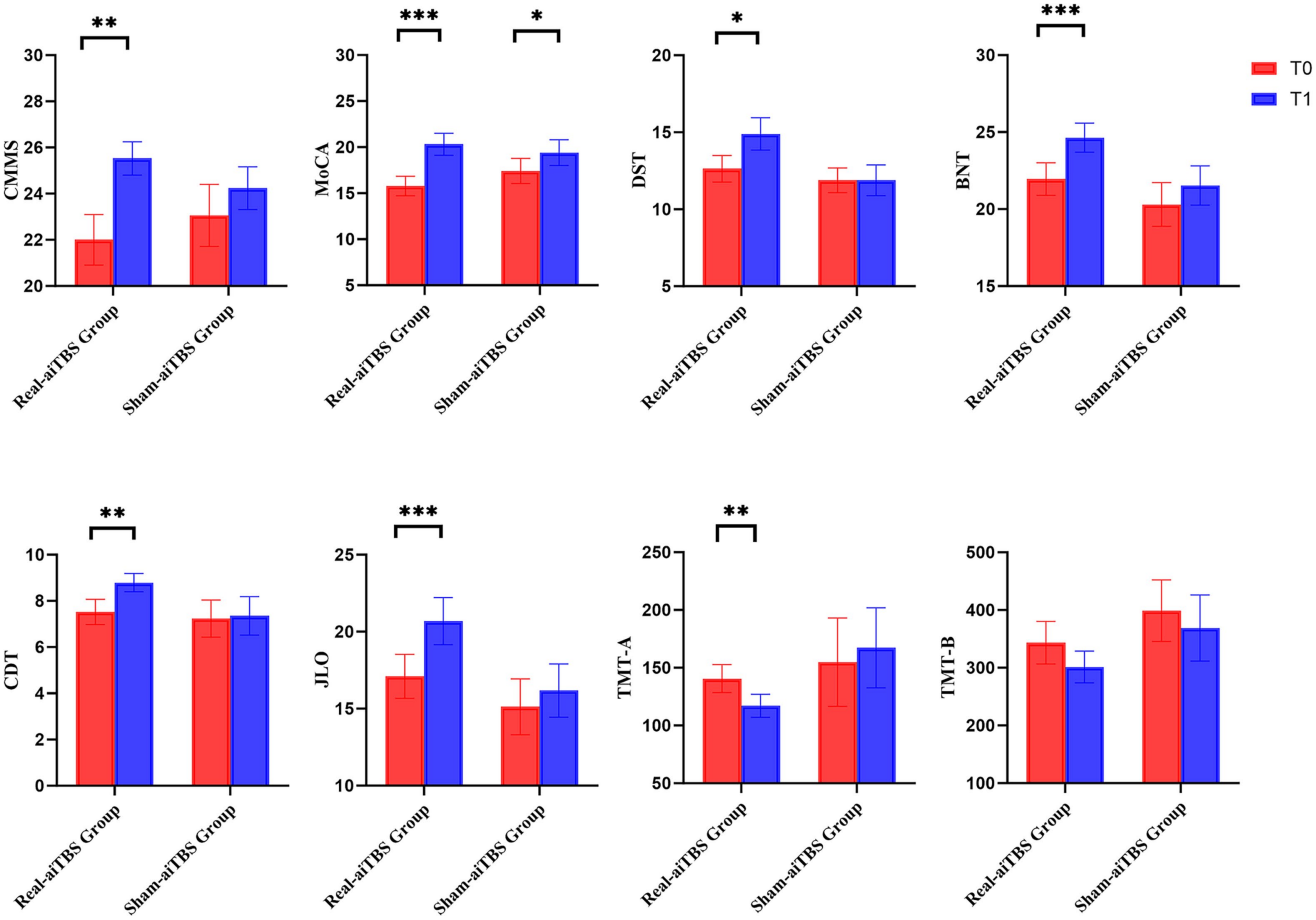
Comparison of cognition scales between two groups. TMT-A = Trail Making Test-A; TMT-B = Trail Making Test-B; DST = Digital Span Test; BNT = Boston Naming Test; CDT = Clock Drawing Test; JLO = Judgment of Line Orientation Test. *p < 0.05; **p < 0.01, ***p < 0.001.

At baseline, cognition function profiles were comparable between the two groups. The administration of 14 real aiTBS sessions yielded significant enhancements compared to baseline in a broad range of metrics: CMMS, MoCA, TMT-A, DST, BNT, CDT and JLO (*P*s < 0.05). With the exception of MoCA score which also showed improvement (*p* < 0.05), no other significant changes were observed in the sham-aiTBS group (Figure 5).

To explore the absence of a significant Group × Time interaction for the MoCA total score, we performed *post-hoc* analyses of its sub-scores. As shown in Tables 5, 6, significant interactions were observed for Visuospatial and Executive Functions (*F* = 5.748, *p* = 0.004, η^2^_p_ = 0.102), Delayed Recall (*F* = 5.172, *p* = 0.007, η^2^_p_ = 0.066), and Attention (*F* = 5.172, *p* = 0.007, η^2^_p_ = 0.093). No significant interactions were found for Naming, Language, Abstraction, or Orientation (*Ps*>0.05).

**Table 5:** Generalized linear mixed model analysis of changes in MoCA subdomain scores from T0 to T2.

MoCA subdomain	Estimated change (T2-T0)		Time effect		Group effect	Group × Time effect		
	Real-aiTBS group Mean [95%CI]	Sham-aiTBS group Mean [95%CI]	*F*-test	*P*-value	*F*-test	*P*-value	*F*-test	*P*-value
Visuospatial and executive functions	1.16 [0.62,1.70]	0.18[−0.43,0.79]	6.984	0.001	10.030	0.002	5.748	0.004
Naming	0.16[−0.02,0.34]	0.41[−0.16,0.99]	4.889	0.009	0.025	0.874	0.971	0.382
Delayed Recall	0.84 [0.38,1.30]	−0.06[−0.52,0.40]	13.218	<0.001	4.125	0.045	3.568	0.032
Attention	1.32 [0.48,2.15]	−0.06[−0.56,0.44]	5.246	0.007	3.726	0.056	5.172	0.007
Language	0.16[−0.33,0.65]	−0.18[−0.50,0.15]	1.744	0.180	0.046	0.831	1.142	0.323
Abstraction	0.95 [0.51,1.39]	0.41 [0.09,0.73]	24.612	<0.001	0.565	0.454	2.699	0.072
Orientation	0.21[−0.52,0.94]	0.06[−0.50,0.62]	2.964	0.056	0.113	0.737	0.091	0.913

**Table 6:** Effect sizes for Group × Time interaction (T2–T0 Change) of MoCA subdomain scores.

Outcome	Partial η^2^	Interpretation
Visuospatial and executive functions	0.102	Medium
Naming	0.019	Small
Delayed recall	0.066	Medium
Attention	0.093	Medium
Language	0.022	Small
Abstraction	0.051	Small
Orientation	0.002	Negligible/very small

For naming capability, the BNT showed a significant improvement in the real-aiTBS group compared to the sham-aiTBS group at T1 (*p* < 0.001). In contrast, the MoCA-Naming subscore revealed no significant Group × Time interaction at either T1 or T2 (*Ps* > 0.05), indicating a lack of detectable naming benefit on this measure.

### 3.5 Affective and autonomic nervous function assessments

**Figure 6:**
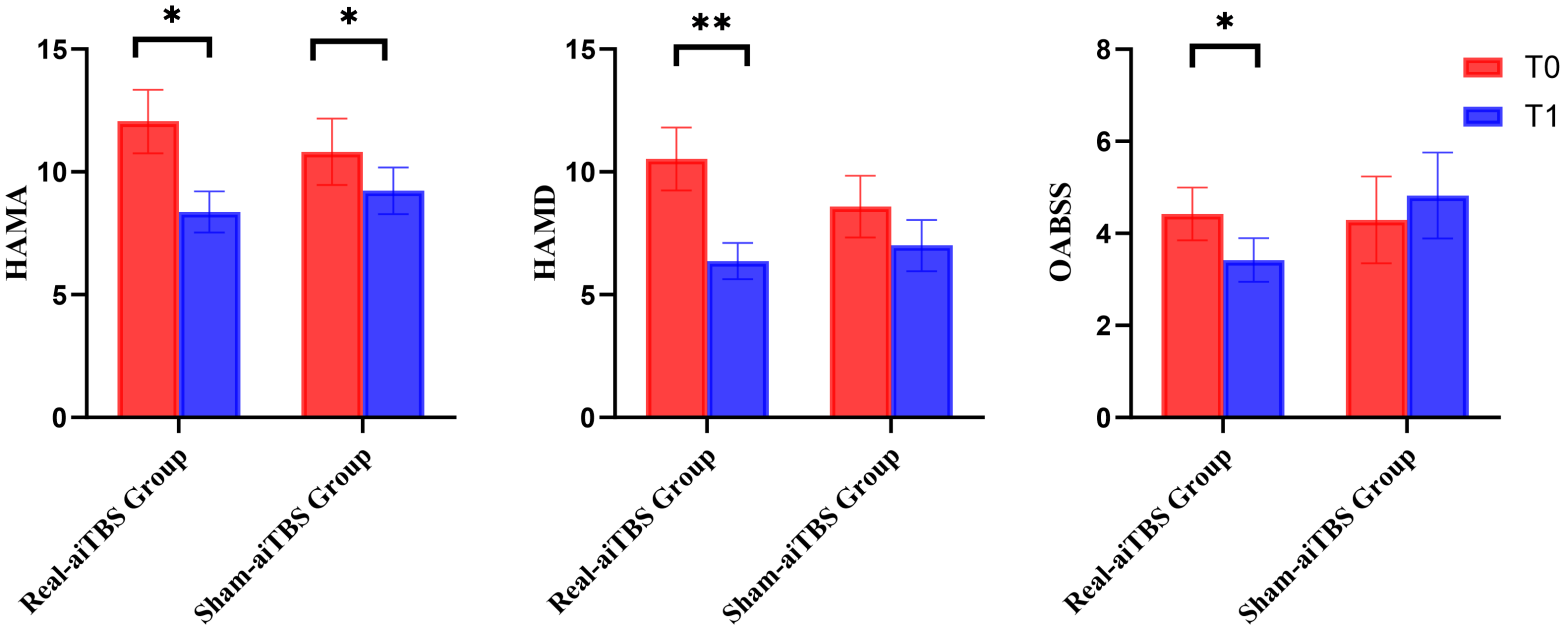
Comparison of affective and autonomic nervous function scales between two groups. HAMA = Hamilton Anxiety Scale; HAMD = Hamilton Depression Scale; OABSS = Overactive Bladder Symptom Score. *p < 0.05; **p < 0.01.

Baseline affective and autonomic function were well balanced between the real and sham groups. Both groups showed significant improvement in the HAMA scores after the intervention compared to before (*p* < 0.05, Figure 6), with a notable decrease in HAMD score following the real-aiTBS (*p* < 0.01, Figure 6). For the autonomic nervous function, the OABSS scale improved significantly after the intervention in the real-aiTBS group (*p* < 0.05, Figure 6).

### 3.6 Correlations between neuroimaging markers and aiTBS response

**Figure 7:**
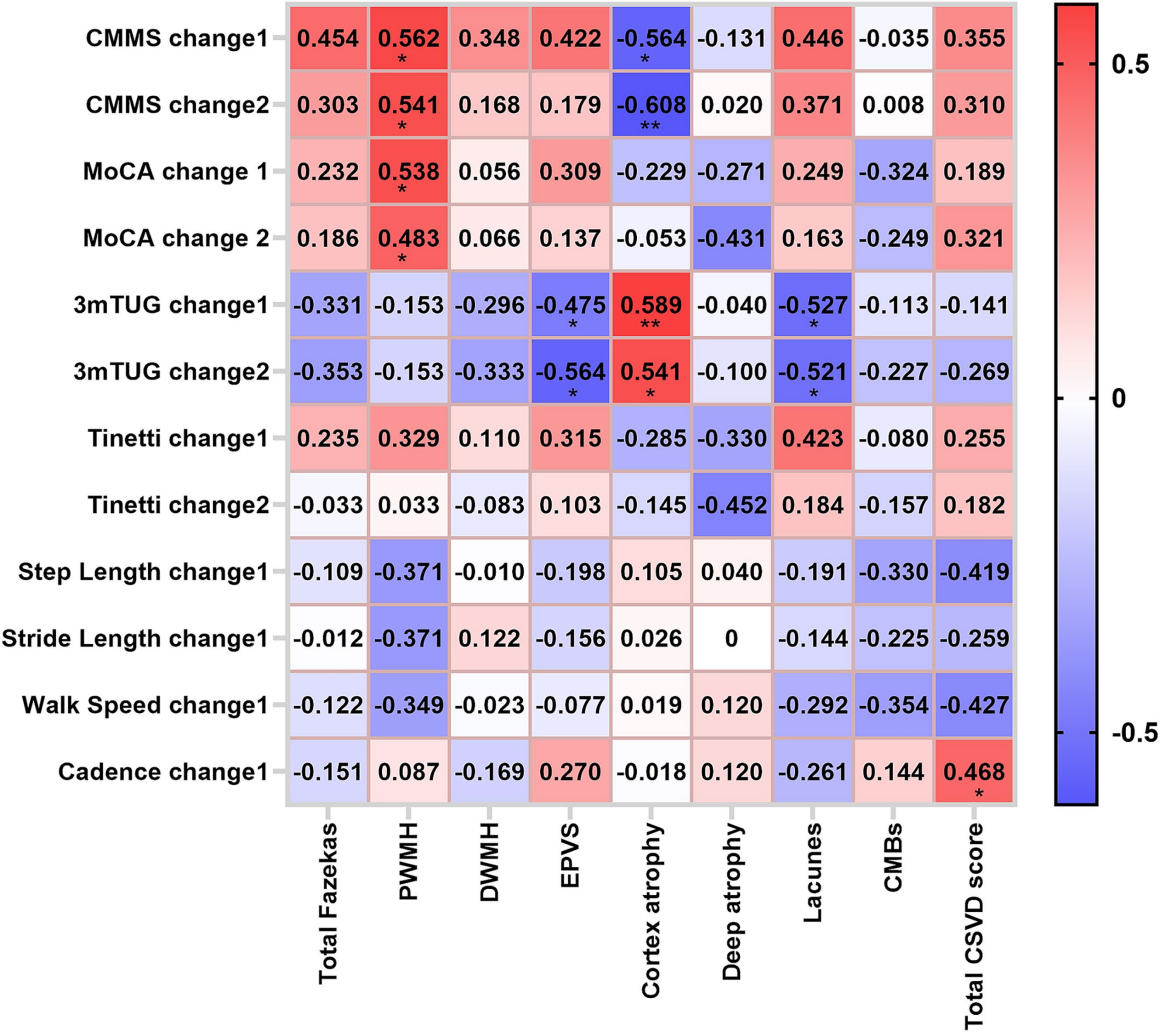
Correlation between neuroimaging markers and post-intervention changes. PWMH, periventricular white matter hyperintensities; DWMH, deep white matter hyperintensities; EPVS, total enlarged perivascular space; CMBs, cerebral microbleeds; Change1: The difference between T1 (final value) and T0 (initial value), calculated as T1 minus T0. Change2: The difference between T2 (final value) and T0 (initial value), calculated as T2 minus T0. *p < 0.05; **p < 0.01.

In the real-aiTBS group, higher periventricular white matter hyperintensity grade was significantly associated with greater cognitive improvement, as reflected by positive correlations with CMMS change (change1: *ρ* = 0.562, *p* = 0.012; change2: *ρ* = 0.541, *p* = 0.017) and MoCA change after aiTBS (change1: *ρ* = 0.538, *p* = 0.018; change2: *ρ* = 0.483, *p* = 0.036). In contrast, total enlarged perivascular space grade showing negative correlations with 3mTUG duration change (change1: *ρ* = −0.475, *p* = 0.040; change2: *ρ* = −0.564, *p* = 0.012). Cortical atrophy is negatively correlated with cognitive improvement (CMMS change1: *ρ* = −0.564, *p* = 0.012; change2: *ρ* = −0.608, *p* = 0.006) and 3mTUG duration improvement (change1: *ρ* = 0.589, *p* = 0.008; change2: *ρ* = 0.541, *p* = 0.017). Lacune burden was significantly associated with greater improvement in 3mTUG duration (change1: *ρ* = −0.527, *p* = 0.021; change2: *ρ* = −0.521, *p* = 0.022). Additionally, total CSVD burden was significantly associated with greater improvement in cadence (change: *ρ* = 0.468, *p* = 0.043). No significant correlations were observed for total white matter hyperintensities, deep white matter hyperintensities, deep brain atrophy, and cerebral microbleeds with gait and cognitive changes (Figure 7).

### 3.7 Safety

During the course of 14 aiTBS sessions among 36 subjects, two subjects (5.56%) reported transient scalp paresthesia during the first session, which resolved spontaneously as the treatment progressed. The remaining 34 subjects (94.44%) reported no notable adverse effects.

### 3.8 *Post-hoc* power analysis

To explore the potential impact of the 28% dropout rate on the risk of Type II error, we performed a post-hoc power analysis based on the actual completers (real-aiTBS group: *n* = 19; sham-aiTBS group: *n* = 17) and the observed effect size for the primary outcome (change from baseline: Cohen’s *d* = −1.659). Using a two-independent-sample *t*-test (two-tailed, *α* = 0.05), the achieved power was >0.999. This indicates that, for the observed effect size, the study had sufficient power despite the dropout. However, because this analysis is *post-hoc* and the outcome definition was refined after reviewing the data, the result should be interpreted with caution. The primary conclusion remains that the treatment effect was large and statistically significant, and the risk of a false-negative finding is low.

## 4 Discussion

As we know, this study is the first randomized controlled trial applying the aiTBS to gait disorder in subjects with CSVD. Here, we demonstrate that significant improvements in multiple dimensions of gait, cognition, affection and autonomic nervous function following real aiTBS compared to sham aiTBS. At follow-up assessments conducted 4 weeks post-intervention, the real-aiTBS group maintained significant improvements in gait performance. At the same time, the side effects of aiTBS were uncommon.

Gait disorder associated with CSVD often manifest as decreased walking speed, shortened stride length, reduced step frequency, widened base of support, increased stride length variability, prolonged double support time, and diminished postural control (2, 37). A growing body of evidence links gait disorder to core neuroimaging markers of CSVD, including white matter hyperintensity, lacune, enlarged perivascular space, and brain atrophy, suggesting that mobility decline reflects cumulative microvascular injury and its downstream network consequences (38–41). Mechanistically, CSVD-related gait disorder is plausibly driven by disruption of distributed motor and cognitive–motor pathways rather than a single focal lesion. As a key node of the motor system, the M1 area initiates and executes motor commands while integrating executive signals transmitted through frontostriatal circuits. Moreover, the corpus callosum supports interhemispheric coordination essential for stable, symmetric gait, and the corticospinal tract—coursing through the corona radiata and internal capsule—conveys descending motor output. The structural damage along these pathways can therefore cause measurable gait slowing and instability (38, 42–45). Neuromodulation targeting the M1 area may influence gait performance by altering corticospinal excitability and reshaping activity across distributed motor-related networks. Given the central role of M1 area in initiating motor output and integrating cognitive–motor information, stimulation of this region can induce downstream modulation along the corticospinal tract and interhemispheric pathways, while concurrently modifying functional connectivity with interconnected regions such as the premotor cortex, primary somatosensory cortex, and parietal areas (23, 46). These network connectivity effects may provide a mechanistic basis for the observed improvements in limb motor function and symptom alleviation (47). In this study, both the 3mTUG duration and the Tinetti scale showed significant Group × Time interactions, they reflect distinct mobility domains. The 3mTUG duration primarily captures global walking speed and transfer ability, while the Tinetti scale assesses balance and gait quality. Our Tinetti subscore analysis also revealed the robust improvement (Supplementary Tables S3, S4), suggesting that aiTBS preferentially improves the qualitative aspects of postural control and stepping pattern. The parallel improvement in 3mTUG duration speed indicates that these qualitative gains also translate into faster performance.

As a hallmark clinical manifestation of CSVD, cognitive dysfunction may involve multiple domains, including executive function, delayed memory, processing speed, language, visuospatial ability, reasoning, and attention (48). We observed that 7-day aiTBS intervention ameliorated cognitive impairment in patients with CSVD. After 14 consecutive sessions of real aiTBS, significant improvements were identified in multiple cognitive domains immediately post-intervention, including comprehensive cognitive ability, visuospatial ability and executive function, immediate memory and attention, as well as naming and graphomotor ability. At the 4-week follow-up after aiTBS, our MoCA subscore analysis revealed that real-aiTBS selectively improved Attention, Delayed Recall, and Visuospatial and Executive Functions – domains directly relevant to gait planning and balance – while leaving other domains unaffected. The MoCA total score, being an average of all subdomains, was therefore diluted by nonresponsive items. Although the BNT showed significant early improvement after real aiTBS, the MoCA-Naming subscore did not reach significance at any time point. This discrepancy likely arises from the BNT’s higher sensitivity to subtle naming changes, rather than a true absence of naming benefit. Clinically, our data suggest that MoCA subscores – particularly Attention and Executive function – are more sensitive outcome measures than the total score. Although M1 area is primarily recognized for motor control, it also contributes to cognitive processes, potentially through modulation of neurotransmission and synaptic plasticity within distributed cortico-subcortical networks (49). Via its extensive connections with the prefrontal and sensory cortex and the basal ganglia, M1 area interfaces with circuits implicated in both motor learning and higher-order cognition (10). Consistent with prior evidence that M1 area-targeted stimulation can facilitate motor learning and may engage the basal ganglia–cortex–thalamus loop, our findings suggest that aiTBS targeting M1 area may confer broader cognitive benefits in CSVD (9, 49, 50). Gait, particularly gait variability, is not merely a motor output but reflects the integrity of higher-order cognitive control networks. Disruption of large-scale brain networks impairs cognitive functions—especially executive function and psychomotor speed—which in turn leads to reduced gait stability and efficiency (51). Peter Mukli et al. also discovered CSVD patients undergoing dual-task assessments, impaired cognitive resource allocation leads to significantly increased gait variability (52). Thus, gait abnormality represent both a consequence and a sensitive functional marker of cognitive network dysfunction (38). The interventions targeting cognitive function may indirectly improve gait performance (53).

Patients with CSVD often experience frequent urination, urgency, and urinary incontinence, and sometimes also fecal incontinence (2). Some anticholinergic drugs may be used to alleviate this symptom, but be limited due to potential side effects (54). TMS induces activation or inhibition of multiple brain networks, altering neural and muscular activity related to urination, and has yielded favorable outcomes in treating urinary dysfunction in patients with multiple sclerosis (55, 56). In this study, the aiTBS was delivered over M1 area in proximity to the paracentral lobule, a region implicated in voluntary bladder control (54). Following stimulation, patients reported symptomatic relief accompanied by improvements in OABSS scores, suggesting that M1 area -targeted aiTBS may modulate frontal–subcortical pathways involved in bladder regulation. Future studies should incorporate more comprehensive autonomic and urodynamic assessments and further explore optimal neuromodulation protocols for urinary and other autonomic dysfunctions in CSVD.

Affective disorder is common in CSVD and typically manifest as depressive and anxiety symptoms. A plausible neurobiological basis involves CSVD-related disruption of fronto-subcortical circuitry, including the prefrontal–subcortical pathways and the corticostriatal–pallidal–thalamocortical loops that regulate mood and emotional control (57, 58). Mechanistically, TMS may exert its effects by promoting neuroplasticity and rebalancing dysfunctional large-scale networks implicated in affect regulation. Our findings are in line with prior reports suggesting that TMS may ameliorate affective symptoms in CSVD (59, 60). At the same time, improvements in gait and cognition may indirectly reduce psychological distress by enhancing functional independence and daily-life participation.

In this study, we implemented an aiTBS protocol with parameters that differ from conventional rTMS, aiming to explore its therapeutic potential in CSVD. Prior evidence suggests that key TBS parameters associated with greater clinical efficacy include relatively lower stimulation intensity, higher pulse dose, and shorter overall treatment courses (61). The stimulation intensity was set at 40% RMT, lower than the 80–120% RMT typically used in conventional rTMS protocols. This choice was based on the heterogeneous and less predictable cortical excitability in CSVD (62–64), safety concerns in older vascular patients (65–67), and evidence that low-intensity TBS remains physiologically active (68, 69). Given that twice-daily sessions over 7 days could lower seizure threshold, we adopted 40% RMT to maintain a robust safety margin while preserving the patterned burst structure. In addition, low-intensity stimulation has been less extensively studied, and its physiological and clinical effects in CSVD remain incompletely characterized. Therefore, our protocol was designed as an exploratory regimen. In many conventional TBS protocols, the pulse number per session is typically around 600. However, in Stanford Neuromodulation Therapy study, each stimulation session comprises 18,00 pulses and ten sessions were applied per day, which indicate that higher pulse doses correlate with superior antidepressant efficacy, while the incidence of side effects remains similar to that of once-daily rTMS (70). These observations provided a rationale for testing a higher-dose, aiTBS strategy in CSVD while maintaining a conservative stimulation intensity. Among 36 participants in this study, 2 subjects (5.56%) reported transient scalp tingling sensations, and no other discomfort or adverse events were documented, indicating the safety of the stimulation parameters.

Our findings also indicate that the therapeutic response to TMS in CSVD patients is strongly modulated by the neuroimaging characteristics of CSVD rather than by overall disease severity alone. Specifically, different CSVD imaging markers appear to reflect distinct pathophysiological substrates (43). Markers such as white matter hyperintensities, enlarged perivascular spaces, and lacunes are generally considered indicators of diffuse microvascular dysfunction and subcortical network disconnection (71, 72). Importantly, these alterations primarily involve disruption of white matter tracts and structure–function coupling, while relatively preserving cortical neuronal connection (73, 74). As a result, patients with more severe periventricular white matter hyperintensities demonstrated greater cognitive improvement, whereas those with more prominent enlarged perivascular spaces, higher lacune counts and total CSVD burden score exhibited more pronounced gait improvement after TMS. This paradoxical association suggests that, in some CSVD phenotypes, functional impairment may remain partially reversible, particularly in neural networks supporting gait and cognition control, providing a larger therapeutic opportunity for TMS (40).

In contrast, cortical atrophy showed a negative association with both gait and cognition improvement after TMS, because of its different pathological nature. Cortical atrophy reflects irreversible neuronal loss, synaptic degeneration, and reduced cortical reserve, which severely constrain compensatory reorganization and treatment effectiveness (75). Unlike subcortical CSVD markers that predominantly affect network connectivity, cortical atrophy directly damages the structural substrate required for functional recovery (75). Together, these findings support a model in which CSVD-related treatment response is neuroimaging phenotype-dependent, with microvascular and subcortical network injuries remaining amenable to TMS, whereas advanced cortical neurodegeneration represents a major limiting factor for CSVD-related functional improvement. This distinction may have important implications for individualized therapeutic strategies and imaging-based patient stratification in CSVD management.

This study has several limitations. First, a significant limitation is the high dropout rate (28%), the possibility of bias due to differential dropout cannot be entirely excluded. Future multi-center studies with larger sample sizes and lower dropout rates are warranted. Second, the study did not conduct multiple or long-term assessments after aiTBS completion. Additional follow-up time points are required, to evaluate the long-term effects of aiTBS. Third, this study did not incorporate post-intervention functional MRI, which limits the ability to verify plastic changes in the M1 region or its network connectivity. Future studies combining TMS with neuroimaging are needed to elucidate the underlying neural mechanisms.

## 5 Conclusion

High-dose aiTBS targeting the M1 area significantly improved gait and cognition function in CSVD patients in the 1 month. After one intervention course, gait performance represented by 3mTUG duration, Tinetti score, fundamental pace characteristics and stride variability all showed measurable improvement. While cognitive functions in the global cognition, executive function, visuospatial ability, memory, attentional capacity, language function, graphomotor ability and affection regulation, voiding function dimensions were also significantly enhanced. The aiTBS intervention is a safe and promising clinical approach in CSVD. The therapeutic response to aiTBS in CSVD patients is related to the CSVD neuroimaging phenotypes. The individualized optimal parameter schemes based on imaging subtypes and long-term efficacy require further research.

## Data Availability

The raw data supporting the conclusions of this article will be made available by the authors, without undue reservation.

## Glossary

CSVD: Cerebral Small Vessel Disease
aiTBS: Accelerated Intermittent Theta Burst Stimulation
3mTUG: 3-meter Timed Up and Go
Tinetti: Tinetti Performance-Oriented Mobility Assessment
CMMS: Chinese version of the Mini-Mental State Examination
MoCA: Montreal Cognitive Assessment
NIBS: Noninvasive brain stimulation
TMS: Transcranial Magnetic Stimulation
iTBS: Intermittent Theta-Burst Stimulation
cTBS: Continuous Theta-Burst Stimulation
RMT: Resting Motor Threshold
HAMA: Hamilton Anxiety Scale
HAMD: Hamilton Depression Scale
TMT-A: Trail Making Test-A
TMT-B: Trail Making Test-B
DST: Digital Span Test
BNT: Boston Naming Test
CDT: Clock Drawing Test
JLO: Judgment of Line Orientation Test
OABSS: Overactive Bladder Symptom Score
rTMS: Repetitive Transcranial Magnetic Stimulation
PWMH: Periventricular White Matter Hyperintensities
DWMH: Deep White Matter Hyperintensities
EPVS: Enlarged Perivascular Space
CMBs: Cerebral Microbleeds
