# Supplementary material for: High-dose accelerated intermittent theta burst stimulation targeting the primary motor cortex for gait and cognitive functions in cerebral small vessel disease: a randomized controlled trial
Source: Front Neurol. 2026 Jun 1;17:1840684. doi: 10.3389/fneur.2026.1840684 (PMC13265494; doi:10.3389/fneur.2026.1840684)
Supplement: Supplementary file 1 [file Table_1.DOCX]

Table S1. Baseline characteristics of completers vs. dropouts

|  | Completers (n=36) | Dropouts (n=12) | Statistic | *P* |
| --- | --- | --- | --- | --- |
|  |  |  |  |  |
| Age, Mean ± SD | 68.58 ± 6.20 | 68.79 ± 7.15 | t=-0.10 | 0.921 |
| Gender (male/female) | 21/15 | 8/6 | χ²=0.01 | 0.939 |
| Education, M (Q₁, Q₃) | 10.00 (9.00, 15.00) | 12.00 (9.00, 12.75) | Z=-0.12 | 0.904 |
| BMI, M (Q₁, Q₃) | 24.95 (23.29, 26.63) | 25.39 (23.30, 26.03) | Z=-0.19 | 0.846 |
| Smoking (%) | 7/29 (19.44) | 2/12 (14.29) | χ²=0.00 | 0.987 |
| Drinking (%) | 8/28 (22.22) | 3/11 (21.43) | χ²=0.00 | 1.000 |
| Stroke (%) | 13/23 (36.11) | 6/8 (42.86) | χ²=0.19 | 0.659 |
| Hypertension (%) | 31/5 (86.11) | 11/3 (78.57) | χ²=0.05 | 0.823 |
| Diabetes Mellitus (%) | 7/29 (19.44) | 2/12 (14.29) | χ²=0.00 | 0.987 |
| Atrial Fibrillation (%) | 1/35 (2.78) | 0/14 (0.00) | - | 1.000 |
| Total CSVD Burden Score(%) |  |  | - | 0.063 |
| 1 | 0 (0.00) | 0 (0.00) |  |  |
| 2 | 3 (8.33) | 0 (0.00) |  |  |
| 3 | 8 (22.22) | 8 (57.14) |  |  |
| 4 | 25 (69.44) | 6 (42.86) |  |  |
