# Supplementary material for: High-dose accelerated intermittent theta burst stimulation targeting the primary motor cortex for gait and cognitive functions in cerebral small vessel disease: a randomized controlled trial
Source: Front Neurol. 2026 Jun 1;17:1840684. doi: 10.3389/fneur.2026.1840684 (PMC13265494; doi:10.3389/fneur.2026.1840684)
Supplement: Supplementary file 2 [file Table_2.DOCX]

Table S2. ANCOVA results for Tinetti change score (T2−T0) with baseline Tinetti as covariate

| Source | Type III Sum of Squares | df | Mean Square | F | Sig. |
| --- | --- | --- | --- | --- | --- |
| Corrected Model | 366.709 | 2 | 183.354 | 71.243 | < 0.001 |
| Intercept | 225.075 | 1 | 225.075 | 87.454 | < 0.001 |
| Tinetti-T0 | 152.835 | 1 | 152.835 | 59.385 | < 0.001 |
| Group | 46.199 | 1 | 46.199 | 17.951 | < 0.001 |
| Error | 84.930 | 33 | 2.574 |  |  |
| Total | 713.000 | 36 |  |  |  |
| Corrected Total | 451.639 | 35 |  |  |  |

Note: R² = 0.812, adjusted R² = 0.801. Dependent variable: Tinetti change score (T2−T0). Tinetti_T0 = baseline Tinetti score.
