# Supplementary material for: High-dose accelerated intermittent theta burst stimulation targeting the primary motor cortex for gait and cognitive functions in cerebral small vessel disease: a randomized controlled trial
Source: Front Neurol. 2026 Jun 1;17:1840684. doi: 10.3389/fneur.2026.1840684 (PMC13265494; doi:10.3389/fneur.2026.1840684)
Supplement: Supplementary file 3 [file Table_3.DOCX]

Table S3. Generalized Linear Mixed Model Analysis of Changes in Tinetti Score Subdomain Scores from T0 to T2

|  | Estimated change (T2-T0) | | Time effect | | Group effect | | Group×Time effect | | |
| --- | --- | --- | --- | --- | --- | --- | --- | --- | --- |
|  | Real-aiTBS group  Mean [95%CI] | Sham-aiTBS group  Mean [95%CI] | F -test | *P*-value | F -test | *P*-value | | F -test | *P*-value |
| Balance | 2.58[1.30,3.86] | -1.47[-3.15,0.21] | 6.434 | 0.002 | 0.740 | 0.392 | | 11.034 | ＜0.001 |
| Gait | 2.95[2.19,3.71] | 1.00[-0.19,2.19] | 33.860 | ＜0.001 | 3.652 | 0.059 | | 6.679 | 0.002 |
