# Supplementary material for: High-dose accelerated intermittent theta burst stimulation targeting the primary motor cortex for gait and cognitive functions in cerebral small vessel disease: a randomized controlled trial
Source: Front Neurol. 2026 Jun 1;17:1840684. doi: 10.3389/fneur.2026.1840684 (PMC13265494; doi:10.3389/fneur.2026.1840684)
Supplement: Supplementary file 4 [file Table_4.DOCX]

Table S4. Effect Sizes for Group × Time Interaction (T2–T0 Change) of Tinetti Score Subdomain Scores

| Outcome | Partial η² | Interpretation |
| --- | --- | --- |
| Balance | 0.179 | Very large |
| Gait | 0.117 | Large |
