# Supplementary material for: High-dose accelerated intermittent theta burst stimulation targeting the primary motor cortex for gait and cognitive functions in cerebral small vessel disease: a randomized controlled trial
Source: Front Neurol. 2026 Jun 1;17:1840684. doi: 10.3389/fneur.2026.1840684 (PMC13265494; doi:10.3389/fneur.2026.1840684)
Supplement: Supplementary file 5 [file Table_5.DOCX]

**Case Report Form (CRF)**

**Demographics and baseline information**

| Participant ID |  | Group | □Real-aiTBS  □Sham-aiTBS |
| --- | --- | --- | --- |
| Age (years) |  | Sex | □ M □ F |
| \| Education (years) \| \| --- \| |  | BMI(kg/m²) |  |
| Clinical history |  | | |
| Medications |  | | |
| \| MRI findings \| \| --- \| |  | | |

**Assessments at T0 (Baseline, Day 0) and T1 (Post‑intervention, Day 7)**

*Note: The same battery was administered at both T0 and T1. Record values in the respective columns.*

| Domain | Assessment tool | T0 value | T1 value |
| --- | --- | --- | --- |
| **Gait & balance** | 3mTUG (seconds) | ______ sec | ______ sec |
|  | Tinetti – balance (/16) | ______ /16 | ______ /16 |
|  | Tinetti – gait (/12) | ______ /12 | ______ /12 |
|  | 3D gait analysis | Data file: Gait_T0_#ID#.xlsx | Data file: Gait_T1_#ID#.xlsx |
| **Cognition** | CMMS (/30) | ______ /30 | ______ /30 |
|  | MoCA (/30) | ______ /30 | ______ /30 |
|  | DST（/38） | ______ | ______ |
|  | CDT（/10） | _____ /10 | ______ /10 |
|  | BNT（number correct/30） | _____ /30 | ______ /30 |
|  | Trail Making Test (TMT) – part A (seconds) | ______ sec | ______ sec |
|  | TMT – part B (seconds) | ______ sec | ______ sec |
|  | Judgment of Line Orientation (JLO) (/30) | ______ /30 | ______ /30 |
| **Emotion** | Hamilton Anxiety Rating Scale (HAMA) (/56) | ______ /56 | ______ /56 |
|  | Hamilton Depression Rating Scale (HAMD) (/52) | ______ /52 | ______ /52 |
| **Overactive bladder** | Overactive Bladder Symptom Score (OABSS) (/15) | ______ /15 | ______ /15 |

**Assessments at T2 (Follow‑up, Week 5, i.e., 4 weeks after T1)**

| Domain | Assessment tool | T2 value |
| --- | --- | --- |
| **Gait & balance** | 3mTUG (seconds) | ______ sec |
|  | Tinetti – balance (/16) | ______ /16 |
|  | Tinetti – gait (/12) | ______ /12 |
| **Cognition** | CMMS (/30) | ______ /30 |
|  | MoCA (/30) | ______ /30 |

**Adverse events | □ None □ Yes (describe): ____________________**

**Intervention adherence | Number of aiTBS sessions completed: ______/ 14**

**Assessor signature | _________________ | Date | _________________**

***The full set of 3D gait parameters for each participant at T0 and T1 is stored in an Excel file following the template provided in Supplementary Gait_Parameters_Template. The template shows the exact data structure (variable names, units, and organization).***
